# Evaluation of the sealing ability of different root canal sealers: a combined SEM and micro-CT study

**DOI:** 10.1590/1678-7757-2016-0584

**Published:** 2018-01-16

**Authors:** Yan HUANG, Kaan ORHAN, Berkan CELIKTEN, Ayşe Işıl ORHAN, Pelin TUFENKCI, Semra SEVIMAY

**Affiliations:** 1 Sichuan University, West China Hospital of Stomatology, State Key Laboratory of Oral Diseases & National Clinical Research Center for Oral Diseases, Chengdu, China.; 2 KU Leuven, University Hospitals Leuven, Faculty of Medicine, Department of Imaging & Pathology, Leuven, Belgium.; 3 Ankara University, Faculty of Dentistry, Department of Dentomaxillofacial Radiology Ankara, Turkey.; 4 Ankara University, Faculty of Dentistry, Department of Endodontics, Ankara, Turkey.; 5 Ministry of Health, 75th Year Ankara Oral and Dental Health Centre, Division of Pedi-atric Dentistry, Ankara, Turkey.; 6 Mustafa Kemal University, Faculty of Dentistry, Department of Endodontics, Hatay, Turkey.

**Keywords:** Scanning electron microscopy, Microcomputed tomography, Root canal, Sealer

## Abstract

**Objective:**

The purpose of this study was to analyze the ability of multiple compounds to seal the dental tubules using scanning electron microscopy (SEM) and micro-computed tomogra-phy (micro-CT).

**Material and Methods:**

Twenty-four single-root human mandibular premolars were selected and instrumented with nickel-titanium rotary file and the final file size was #40/06. They were then randomly allocated into 2 groups, and all samples were filled with single cone gutta-percha (#40/06) and one of the tested sealers (AH Plus and EndoSequence BC sealers). All specimens were scanned using micro-CT and then three from each group were randomly selected for SEM analysis.

**Results:**

According to SEM, both root canal sealers showed sufficient adaptation to dentin along the whole length of the root canal, though the coronal sections presented superior sealing than the apical sections. Micro porosity analyses revealed that the volume of closed pores and the surface of closed pores had the largest values in the coronal sections, followed by the middle and the apical sections for both sealants (p<0.05). However, no significant difference was observed for those two parameters between AH Plus and EndoSequence BC sealers in any of the three sections (p>0.05), whereas they were larger in the apical section when the AH Plus sealer was used.

**Conclusions:**

By using the single cone technique, neither EndoSequence or AH Plus pro-vides a porosity-free root canal filling. The EndoSequence BC sealer may have similar sealing abilities regarding the whole root canal as the AH Plus sealer. A better sealing effect could be obtained in the coronal and middle sections of a root canal than the apical part by using the tested sealers.

## Introduction

The long-term success of endodontic therapies relies on complete filling after root canal obturation^12^. Microleakage is one of the significant causes for endodontic failure, which occurs due to poor contacts between the gutta-percha and the sealer, the sealer and the dentin, or through voids within the sealer^16^ ^,^ ^26^. In general, the oral bacteria could contaminate the entire length of root canal within 30 days of obturation^16^, while endotoxins from *Actinobacillus actinomycetemcomitans* could be observed in obturated root canals within 20 days^26^.

Due to the complexity of root canal systems, pulp tissue and inorganic debris remain in areas instruments and irrigation solutions cannot easily access after root canal treatments. Thus, microorganisms surviving in the root canal will subsequently grow and spread to the periradicular areas between the sealer and dentin^27^. Permanent coronal restorations also provide seals equally as important as the apical seal after the root canals are filled^3^. When insufficient coronal sealing occurs or the root canal remains open (e.g., when sealing is delayed for permanent fillings, broken fillings, or secondary caries formation; etc), oral bacteria will access the apical foramen^28^.

It is not easy to achieve a complete filling with the current root-filling materials used in the clinic, due to the dimensional changes and lack of adhesion from gutta-percha, which is also the reason to use endodontic sealers in combination of gutta-percha. Thus, the adaptability of a sealer to the dentin is the primary factor influencing microleakage and reinfection of the root canal^19^. Many endodontic sealers are used in clinical practice, including the recently-introduced calcium silicate-based sealers. The EndoSequence BC sealer (Brasseler USA, Savannah, Georgia, USA; also named the iRoot SP; (Innovative. BioCeramix Inc, Vancover, Brtish Columbia, Canada) has been introduced as an ideal premixed and injectable biomaterial in the clinical, exhibiting excellent radiopaque, zero-shrinkage, insoluble, and hydrophilic (using moisture from the dentinal tubules to initiate and complete its setting reaction) characteristics^9^.

The adaptation of a sealant to the dentin has generally been evaluated using stereo-microscopy, confocal laser microscopy, scanning electron microscopy (SEM), leakage tests, and digital imaging^13^ ^,^ ^20^ ^,^ ^25^. Compared to other two-dimensional (2D), time-consuming, and destructive evaluation ways, micro-computed tomography (micro-CT) is one kind of advanced imaging modality used to scan filled roots and reconstruct them three-dimensionally (3D) for the assessment of the sealant's adaptation to the root canal walls^13^. To the best of our knowledge, however, no research has been performed on 3D micro porosity by using micro-CT to assess the sealing ability of BC sealer in the whole root canal system and its circumferential dentin area. Moreover, there is no study yet using SEM and micro-CT for this purpose.

Therefore, the aim of this study was to quantitatively evaluate and compare the sealing ability of BC sealer and AH Plus at the apical, middle, and coronal dental tubules using SEM and micro-CT. The study was performed under the null hypothesis that no differences in the ability of sealing dental tubules would be observed between the two tested root canal sealants.

## Material and methods

In this study, similarly sized, single-rooted, human mandibular premolars, extracted by orthodontic reasons, were collected from patients in the clinic, after their verbal informal consent for the use of these teeth in the lab with the ethical approval from the Ethics Committee of Mustafa Kemal University (No. 20012017/4919). All teeth were decontaminated in 5.25% sodium hypochlorite for 2 hours. They were then stored in distilled water until further testing. Teeth were examined using an operating microscope (Carl Zeiss Meditec AG, Oberkochen, Germany) at 20× magnification, and those with immature apices, caries, restorations, fractures, or cracks were excluded from the study, and only teeth with oval canals were included in the standardization procedure for the experiment. Preoperative radiographs were obtained in the mesiodistal and buccolingual directions to confirm the presence of a single unmanipulated root canal without root caries, resorption, or calcification. In total 24 teeth were included and decoronated at the cemento-enamel junction, and each root was adjusted to about 12 mm in length. Subsequently, a #10 K-File (Dentsply Maillefer, Ballaige, Switzerland) was inserted into the root canal until the tip was at the apex. The working length was determined by subtracting 0.5 mm from this length.

**Figure 1:**
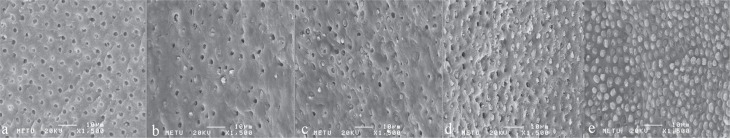
Scanning electron microscopy (SEM) images showing (a) open dentin tubules after EDTA treatment; (b) partially obturated dentin tubules in the apical section using AH Plus; (c) partially obturated dentin tubules in the apical region using the EndoSequence BC sealer; (d) superior sealing in the coronal region using AH Plus; and (e) superior sealing in the coronal region using EndoSequence BC sealer

All samples had a taper of 0.06. Accordingly, all teeth were instrumented to a size of 40/06 using a crown-down technique with an EndoSequence 0.06 taper NiTi rotary instrument (Brasseler). Irrigation was performed with 2 mL 2.5% NaOCl between each instrument use. Following tooth manipulation, a final 1 min rinse with 2 mL 2.5% NaOCl, 2 mL 17% EDTA (Ethylenediaminetetraacetic Acid, Patterson Dental Supply, Fort Worth, Texas, USA), and 10 mL distilled water was performed to eliminate the smear layer. Canals were then dried with paper points (Dentsply Tulsa Dental, Johnson City, Tennessee, USA), and those samples were randomly divided into two groups (n=12 for each group). After the micro-CT tests, three teeth from each group were randomly selected for the evaluation of the smear layer using SEM (Figure 1).

Root canal sealants were prepared according to the manufacturers' instructions and inserted inside the canal by a size 40 lentulo spiral (Produits Dentaires SA, Vevey, Switzerland) for evenly distributing sealers in the whole canal together with the single cone technique. Group 1: teeth were filled with AH Plus Root Canal Sealer (Dentsply DeTrey, Konstanz, Germany) and 40/06 gutta-percha Dentsply Maillefer, Ballaiges, Switzerland); Group 2: teeth were filled with EndoSequence BC Sealer (Brasseler USA, Savannah, Georgia, USA; and 40/06 gutta-percha (Dentsply Maillefer, Ballaiges, Switzerland). After the filling, the roots were stored at 37°C and 100% humidity for 5 days to allow the sealer to set entirely. The sealer volume and application followed steps previously described^11^.

### Micro-CT imaging acquisition

Scanning was performed using high-resolution micro-CT Skyscan 1172 (Brüker, Kontich, Belgium) at 100 kVp, 100 mA beam current, 0.5 mm Al/Cu filter, 13.67 μm pixel size, 0.5 step rotation, and 30% beam hardening. To minimize ring artefacts, air calibration of the detector was performed prior to each scan. Each sample was rotated 360° with an integration time of 5 min. Additional settings were also implemented, including a beam-hardening correction as previously described, and optimal contrast limits (0–0.06) based on prior scanning and reconstruction of the teeth. For visualization and quantification of 1,000 × 1,000 pixel two-dimensional (2D) axial images, NRecon software (ver. 1.6.7.2; Brüker, Kontich, Belgium) was used with an algorithm described by Bouxsein, et al.^5^ (2010). For the reconstruction, the smoothing was initially set to zero, followed by a setting of 40% when the ring artefact correction (flat field correction) was applied. The contrast limits were set according to the Skyscan instructions.

### Micro-CT imaging analysis

From the reconstructed micro-CT images, the roots were divided into apical (0–4 mm), middle (4–8 mm), and coronal (8–12 mm) sections. For micro porosity analyses, a fixed ring area (diameter of 2 mm) was selected as region of interest (ROI) along the different sections of tooth using CTAn software (version 1.12.9, Brüker, Kontich, Belgium), which included the dentin, root canal sealant, and the guttapercha. All software parameters and the magnification, contrast, and imaging enhancement tools were kept the same to analyze the 3D microarchitectures of each sample. Once the appropriate ROI (in 3D volume) was selected, binary images were obtained and an appropriate greyscale threshold was manually selected to distinguish the gutta-percha, root canal sealant, and dentin. Despeckling was performed to remove white speckles in the 3D images that were fewer than 10 voxels. Following this, 3D imaging analyses were performed to calculate the porosity of the sealant within the ROI volume.

### Scanning electron microscopy evaluation

After micro-CT scanning, three teeth from each group were randomly selected for SEM analysis. Roots were sectioned longitudinally in the labiolingual direction and divided into apical (0–5 mm) and coronal (7–12 mm) sections. Sections were vacuum dried, coated with gold, and then examined by SEM (Carl Zeiss NTS GmbH, Oberkochen, Germany). The penetration of sealants into the dentinal tubules and adaptation of each sealant to the dentin were examined from the coronal to apical ends at 1,500×magnification, and finally, the microphotographs were taken.

### 3D micro porosity analysis

In this study, the total ROI volume (mm^3^), object volume (dentin volume, mm^3^), volume of closed pores (mm^3^), surface of closed pores (mm^2^), volume of open pores (mm^3^), and open porosity (%) were measured using CTAn software (version 1.12.9, Brüker, Kontich, Belgium).

**Figure 2:**
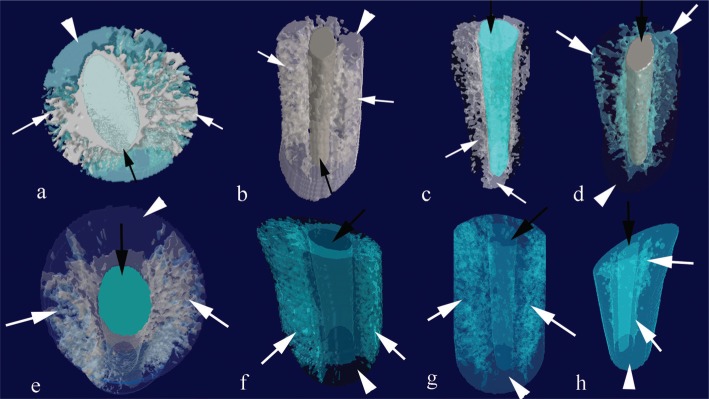
Micro-computed tomography (micro-CT) images showing a three-dimensional (3D) representation of AH Plus (a) and the EndoSequence BC sealer (e) in the coronal sections from an axial view. The superior penetration could be observed in the coronal (b-AH Plus, f-BC sealer), middle (c-AH Plus, g-BC sealer), and apical sections (d-AH Plus, h-BC sealer). White arrows indicate root canal sealers; black arrows indicate the gutta-percha; and the arrowheads indicate the dentin

An open pore was defined as the pore that intersects the boundary of the ROI, which means that an open pore was connected to the outside in 2D or 3D. Therefore, open pores were a property of the ROI, where the root canal sealant penetrated into dentinal tubules and connected from the inside to the outside of the ROI. In contrast, a closed pore was the one without connecting to the outside in 2D or 3D. Closed pores were viewed as black pixels surrounded by a border of white pixels. Such pores were taken as dentin tubules already filled or sealed. The open porosity was calculated as the volume of open pores (as defined above) as a percentage of the total ROI volume. Following those calculations, CTVol software (version 2.2.3.0; Brüker, Kontich, Belgium) was used to generate a 3D model of each sample and assess the distribution of open and closed pores. From the 3D models, the dentin volume, the root canal volume penetrated by the sealant, and the total ROI volume were calculated. As shown in Figure 2, each segmentation was assigned different colors using CTVol software.

### Statistical analysis

The differences between the two groups were assessed using the two independent-sample t-test and the Mann-Whitney *U*-test while the normality was not met. SPSS statistical software (ver. 20.0, Chicago, IL) was used for all analyses, and p values <0.05 were considered statistically significant.

## Results

SEM analyses of root canals obturated with tested root canal sealers revealed that their adaptation to dentin was sufficient along the length of the root canal. Compared to the apical section, the coronal sections showed superior sealing where the texture of the sealers in the tubules was homogeneous (Figure 1).

Micro-CT testing showed a clear overview of tooth dentin, sealant materials, and gutta-percha at assigned grey values. The micro pores at the interface could be observed between the root canal dentin and the sealer filling in all tested groups. The 3D structural parameters of micro pores in the whole root canal system using micro-CT were summarized in Table 1. The volume of closed pores was larger for the AH Plus sealant in the apical section compared to the EndoSequence BC sealer, though without statistical significance. The volume of closed pores and the surface of closed pores showed the largest values in the coronal sections, followed by the middle and the apical sections for both sealants (p<0.05). However, no significant difference was observed for the volume of closed pores and the surface of closed pores between AH Plus and BC sealers in all three sections (p>0.05), whereas they were larger in the apical section when the AH Plus sealant was used.

**Table 1:** Three-dimensional (3D) structural parameters of micro pores in the whole root canal system investigated by micro-CT

Sections	Groups	Number of samples (N)	Total ROI Volume (mm3)	Object (Dentine) Volume (mm^3^)	Volume of Closed Pores (mm^3^)	Surface of Closed Pores (mm^2^)	Volume of Open Pores (mm^3^)	Open Porosity (%)
Apical Third	AH Plus Sealer	12	7.177	1.500	0.151^a^	0.231^a^	4.49^a^	64.24^a^
	BC Sealer	12	7.400	1.420	0.115^a^	0.189^a^	6.24^c^	73.86^a^
Middle Third	AH Plus Sealer	12	7.235	1.445	0.354^b^	0.322^b^	6.10^b,c^	70.24^a^
	BC Sealer	12	7.960	1.330	0.408^b^	0.431^b^	6.84^d^	83.25^a,b^
Coronal Third	AH Plus Sealer	12	7.383	1.358	0.620^c^	0.636^c^	6.32^b,c^	75.39^a,b^
	BC Sealer	12	7.962	1.330	0.861^c^	0.763^c^	6.72^d^	82.74^b^

Same superscript letters indicate no statistical difference (p>0.05).

Different superscript letters indicate statistical significance (p<0.05).

As shown in Figure 2, the BC sealer had a larger volume of open pores than the AH Plus (p<0.05), no matter which section of teeth. The volume of open pores was also larger in the coronal section compared to the apical section for both sealants (p<0.05). Similarly, the open porosity was also larger in the coronal section compared to the apical section for both sealers, though significance only existed for the BC sealer (p<0.05); however, no significant difference was observed between the coronal and middle sections (p>0.05).

## Discussion

Hermetic sealing is the primary factor associated with the success of root canal treatment, and Ingle, et al.^4^ (2008) pointed out that 58% of treatment failures were due to incomplete obturation. Thus, decontamination and 3D obturation are essential following root canal treatments^21^. Because of this, root canal filling materials are continually improving, and bioactive materials are becoming increasingly popular. The EndoSequence BC sealer is one of the ideal bioactive sealants that contains nanoparticles (about 2 μm in diameter) facilitating the penetration into dentinal tubules^1^. Previous studies suggested that AH Plus can be considered the gold standard for root canal sealants; therefore, we compared this sealant to the bioactive EndoSequence BC sealant^3^.

Obturation quality may be influenced by the morphology of the root canal (round or oval)^24^. For this reason, we only selected the oval canal that facilitated the operation of the microscope. The final irrigation solution of 17% EDTA was applied for 1 min to effectively open the dentin tubules. The low surface tension of EDTA also facilitates its access into the dentin tubules to remove the smear layer^29^, which improves access and adaptation of the sealants to the dentin^7^. However, it has to be noted that because of applying EDTA, the open porosity in the whole root canal system - especially in the coronal section - could be increased by removing smear layers.

Micro porosity analyses revealed that the volume of closed pores and the surface of closed pores had the largest values in the coronal sections and the smallest in the apical sections for both sealants in the entire levels of root canal. This was also confirmed by our SEM observation. These results were consistent to another marginal adaptation study^23^, which also showed that the coronal sections had superior adaptation compared to apical sections. On the other side, the volume of open pores from BC sealer were significantly larger than the AH Plus sealant, while for the open porosity, the differences between two sealers vanished, suggesting that BC sealer may have more penetration into the dentin tubules. This may be due to the small particle size of the EndoSequence BC sealer (about 2 μm in diameter) or the viscosity of the calcium phosphate silicate ceramic-based materials that facilitate the flow of the sealant into the dentinal tubules.

Bioceramic materials contain alumina, zirconia, bioactive glass, glass ceramics, hydroxyapatite, and calcium phosphates^17^. The alkaline nature of bioceramic by-products has been reported to denature collagen fibers, which facilitates the penetration of sealers into the dentin tubules^2^. However, AH Plus is naturally acidic, which may limit its bonding to dentin. Moreover, AH Plus contains a polymer that contracts upon polymerization, which may result in sealant cracking and deterioration. Thus, it is likely for these reasons that the EndoSequence BC sealer shows superior sealing ability than AH Plus, but has yet to be confirmed by further in-vivo follow-up studies.

In the current study, a similar volume of closed pores was observed between the EndoSequence BC sealer and the AH Plus, which indicated that tested sealers adapted or penetrated equally to the dentin in the coronal, middle, and apical sections. Similarly, using a fluid filtration method and SEM, Zhang, et al.^30^ (2009) investigated the sealing ability of the iRoot SP sealer and the AH Plus sealer to the apical section of teeth roots. It was found that the iRoot SP using the single-cone technique and the AH Plus using the continuous wave condensation technique were equivalent in fluid leakage. SEM also revealed that both sealers provided gap-free and gap-containing regions within the canals. Consistent with those findings, the SEM observation in this study showed that the apical adaption of the EndoSequence BC and AH Plus sealants were also similar. However, Al-Haddad, et al.^1^ (2015) reported that the EndoSequence BC sealer was significantly thicker than AH Plus or MTA Fillapex (mineral trioxide aggregates) by using the lateral compaction technique, which improved the sealing ability. The possible reason for this discrepancy could be derived from the different obturation techniques and that the results from this study were based on the single-cone technique, which was proved to be an effective way obturating well-tapered root canals after adequate rotary instrumentation^18^.

The degree of adhesion of the sealer to the dentin wall depends largely on the intermolecular surface energy and cleanliness of the dentin, as well as the surface tension and wetting ability of the sealant. Dentin at the coronal, middle, and apical sections has different surface energies and cleanliness. Cleanliness is an important factor for sealer adaption, which could be difficult to achieve in the apical region due to difficulties in removing the smear layer. The smear layer often blocks sealer entry to the dentin tubules, and it was suggested that differences between the apical and coronal regions may be due to the lower density and diameter of dentin tubules in the apical regions^10^. This suggestion may also explain the increased porosity of the coronal and middle regions - shown as the volume of open pores - compared to those observed in the apical region in our study.

High-resolution micro-CT is a nondestructive, highly accurate method of imaging that is becoming increasingly used for the noninvasive assessment of 3D microstructures. In addition to measuring volume, micro-CT facilitates qualitative analyses of images and differentiates between filling materials, voids, and tooth structures, based on the imaging grayscale^8^ ^,^ ^15^. Additionally, it is found that the root-filling materials and the voxel resolution applied during scan can influence the presence of artifacts and thus the observed results^6^. Thus, it is preferable to apply smaller voxel size and FOV for minimizing the presence of artifacts and improving the diagnostic or evaluation accuracy in root filled teeth. As pointed by another study^22^, a resolution of 34 and 68 μm would be sufficient for endodontic micro-CT studies with respect of root anatomy. The resolution of micro-CT used in the current study is as high as 13.67 μm voxel size and the grayscale-based imaging segmentation, making it possible to differentiate a clear porosity structure and further assess their dimensions in the sealer region.

So far yet, none of the previous work on root sealant has focused on the characterization of either open or closed porosity. This study shows the ability of micro-CT to observe open- and closed-pore size, surface, and location in the interface between dentin and sealants in 3D. However, while assessing micro-CT images from endodontic cases, it is necessary to consider beam-hardening effects. As the result of the poly-chromaticity of the X-ray source, this imaging artefact can cause visual distortions of the reconstructed objects such as edge enhancement of the root-filling materials. To compensate these artefacts, a beam-harding correction was performed during imaging reconstruction, which inevitably lead to a decrease of the image quality at certain level. Moreover, one should be aware of the voxel size limitation of micro-CT because of its inability to analyze the resulting smear layer and debris retained in the dentin tubules; SEM can be used to expatiate the surface morphology of root canals, enabling the confirmation of filling materials presented on the root canal wall. Thus, the combination of SEM and micro-CT analyses can be a powerful approach for the studies assessing the sealing ability^14^.

In summary, within the limitation of the study, the null hypothesis that no differences would be observed between the abilities of EndoSequence and AH Plus to seal dentin tubules is accepted while using the single cone technique, which suggests that EndoSequence BC sealer has a similar sealing ability in the entire root canal as the AH Plus sealer does. A better sealing effect could be obtained in the coronal and middle sections than the apical part by using any of those tested sealers.
